# Baculovirus Vectors Induce the Production of Interferons in Swine: Their Potential in the Development of Antiviral Strategies

**DOI:** 10.3390/vetsci8110278

**Published:** 2021-11-17

**Authors:** Guido Nicolás Molina, Sabrina Amalfi, Ignacio Otero, Oscar Taboga, María Paula Molinari

**Affiliations:** 1 Instituto de Agrobiotecnología y Biología Molecular (IABIMO), Instituto Nacional de Tecnología Agropecuaria (INTA), Consejo Nacional de Investigaciones Científicas y Técnicas (CONICET), Hurlingham, Buenos Aires B1686IGC, Argentina; molina.guido@inta.gob.ar (G.N.M.); amalfi.sabrina@inta.gob.ar (S.A.); taboga.oscaralberto@inta.gob.ar (O.T.); 2 Centro de Ciencias Veterinarias (CCV), Universidad Maimónides (UMAI), Buenos Aires B1686IGC, Argentina; otero.ignacio@maimonides.edu

**Keywords:** baculovirus, interferon, swine, antiviral

## Abstract

The huge variety of viruses affecting swine represents a global threat. Since vaccines against highly contagious viruses last several days to induce protective immune responses, antiviral strategies for rapid control of outbreak situations are needed. The baculovirus *Autographa californica* multiple nucleopolyhedrovirus (AcMNPV), an insect virus, has been demonstrated to be an effective vaccine vector for mammals. Besides the ability to display or transduce heterologous antigens, it also induces strong innate immune responses and provides IFN-mediated protection against lethal challenges with viruses like foot-and-mouth disease virus (FMDV) in mice. Thus, the aim of this study was to evaluate the ability of AcMNPV to induce IFN production and elicit antiviral activity in porcine peripheral blood mononuclear cells (PBMCs). Our results demonstrated that AcMNPV induced an IFN-α-mediated antiviral activity in PBMCs in vitro. Moreover, the inoculation of AcMNPV in piglets led to the production of type I and II IFNs in sera from inoculated animals and antiviral activities against vesicular stomatitis virus (VSV) and FMDV measured by in vitro assays. Finally, it was demonstrated that the pseudotyping of AcMNPV with VSV-G protein, but not the enrichment of the AcMNPV genome with specific immunostimulatory CpG motifs for the porcine TLR9, improved the ability to induce IFN-α production in PBMCs in vitro. Together, these results suggest that AcMNPV is a promising tool for the induction of IFNs in antiviral strategies, with the potential to be biotechnologically improved.

## 1. Introduction

Despite the efforts to develop new vaccines against swine infectious diseases, porcine viruses remain a serious threat for animal health and food safety and cause serious economic losses [1,2]. Nevertheless, the urgent need for new and inexpensive antivirals for disease control in pigs is still unmet. Interferons (IFNs), the main antiviral soluble factors of innate immunity, have been thoroughly studied and even used for the treatment of several human and animal viral diseases [3,4,5,6]. Although their direct use in swine and other livestock animals is not profitable, IFN-based antiviral strategies could be promising against epidemic IFN-sensitive viruses like porcine reproductive and respiratory syndrome virus [7], porcine epidemic diarrhea virus [8], African swine fever virus [9], and influenza virus [10,11].

IFN-based antiviral strategies have been mainly studied for the control of foot-and-mouth disease (FMD). FMD inactivated vaccines are serotype- and subtype-specific and require seven days approximately to induce a protective humoral immune response [12], leaving a window of susceptibility. This, together with the high contagiousness and the short incubation times of FMD, makes the development of new antiviral strategies to induce fast protection crucial, especially during outbreak situations. The group of Dr. Grubman has demonstrated that the delivery of IFN genes by replication-defective adenovirus Type 5 (Ad5) induces fast protection against foot-and-mouth disease virus (FMDV) in pigs [13,14,15,16,17]. Certain DNA or RNA molecules are also being studied with this aim, because they can trigger the expression of IFNs in vivo after impacting on cytosolic nucleic acid sensors or endosomal toll-like receptors [3,18,19,20,21]. However, the impact on several of these pattern recognition receptors depends, among other aspects, on the particular sequence [22], and the entry and intracellular trafficking of the molecules [19,23,24,25].

Baculoviruses, a family of viruses that infect insects [26], are used as vectors for gene delivery in vertebrate cells, among other applications. Their use has been particularly studied in depth in mammals, and modifications like the pseudotyping of baculoviruses with the protein G from the vesicular stomatitis virus (G-VSV) have been developed to increment the tropism and the transduction efficiency [27,28,29]. Baculoviruses deliver their double-stranded DNA genomes not only to the nucleus, but also to certain endosomal compartments where their CpG motifs can be sensed as well as to the cytoplasm in mammalian cells without any detectable replication [29,30,31,32,33,34,35,36]. In this way, it has been shown that they can impact the TLR9/MyD88-dependent pathway [37,38,39,40] as well as the STING-dependent cytosolic DNA-sensing pathway [29,36,39] in mice, leading to the production of pro-inflammatory cytokines and IFNs within 12 h after inoculation [35,39,41,42].

Many research groups have taken advantage of the strong immunostimulatory properties of the baculoviruses and successfully used them as vaccine vectors that express or display heterologous antigens [42,43,44,45,46]. Besides the numerous advantages that the use of baculovirus has as a vaccine vector, the administration of baculoviruses in mice establishes a rapid nonspecific antiviral status that provides protection against challenges with porcine viruses like encephalomyocarditis virus [47] and influenza A virus [43]. Our group has previously demonstrated that the baculovirus AcMNPV also protects C57Bl/6 mice against a lethal challenge with foot-and-mouth disease virus (FMDV) from 3 h and up to 3 days after inoculation, totally abrogating the onset of the disease [48,49]. In addition, we have recently shown that this antiviral status is mediated mainly by type I IFNs, although NK/NKT cells, the main producers of type II IFNs, are also important to prevent the onset of the disease [50].

Despite some research groups having demonstrated that baculovirus vectors can be used to induce adaptive immune responses against heterologous viral antigens in swine [51,52,53,54,55], their ability to induce the production of IFNs by porcine immune cells in the short term has never been studied before. Since baculoviruses could have distinct immunomodulatory capacities in mice and other hosts, the purpose of this work was to study the potential of AcMNPV to induce a rapid production of IFNs, especially those of type I, in porcine mononuclear cells. Firstly, the impact of the baculovirus AcMNPV on the production of IFNs in peripheral blood mononuclear cells (PBMCs) was evaluated in vitro and later, piglets were intravenously inoculated to assess the ability of the vector to induce these cytokines with antiviral activity in vivo. Additionally, the effect of molecular approaches to optimize the induction of type I IFNs in PBMCs by enhancing the interaction between innate sensors and AcMNPV was analyzed: the addition of specific immunostimulatory CpG sequences for the porcine TLR9 into the AcMNPV genome and the pseudotyping of virions with G-VSV.

## 2. Materials and Methods

### 2.1. Cells and Viruses

Budded virions of the baculovirus model species AcMNPV, or the recombinant baculoviruses AcMNPV enriched with porcine CpG motifs (Ac-pCpG) and pseudotyped with the protein G from VSV (Ac-GVSV), were obtained by the Bac-to-Bac system (Invitrogen, Thermo Fisher Scientific, Buenos Aires, Argentina). These viruses were amplified in Sf9 cells (ATCC CRL-1711) cultured in Grace’s medium with 10% of fetal bovine serum (FBS) at 27 °C and viral titers calculated as described before [50]. Supernatant from non-infected Sf9 cells was also collected to be used as a mock of infection.

For antiviral assays, vesicular stomatitis virus (VSV) serotype Indiana (kindly provided by Dr. Luis Scolaro, School of Exact and Natural Sciences, University of Buenos Aires, Argentina) and FMDV strain O1/Campos (provided by the national sanitary authority SENASA) were amplified and titrated by end-point dilution in Vero cells (ATCC CCL-81) and BHK-21 cells (ATCC CCL-10), respectively. As required, VSV and FMDV were handled under biosafety level II conditions or in the Laboratory of Biosafety Level 4 (BSL4-OIE) of the Institute of Virology and Technological Innovation of the National Institute of Agricultural Technology (INTA-CONICET), respectively.

The bovine kidney cell line MDBK (ATCC CCL-22) and the porcine kidney cell line LFBK [56] used in the antiviral assays were kindly provided by the Cell Culture Laboratory from CICVyA (Centro de Investigación en Ciencias Veterinarias y Agronómicas, INTA) and Dr. Mariano Perez Filgueira from IVIT (Instituto de Virología e Innovaciones Tecnológicas, INTA-CONICET) respectively. These cells were cultured in Dulbecco’s modified Eagle’s medium (DMEM) (Gibco) supplemented with 10% FBS (Internegocios S.A., Mercedes, Buenos Aires, Argentina) and antibiotic-antimycotic solution (Gibco, Thermo Fisher Scientific, Buenos Aires, Argentina) at 37 °C and 5% CO_2_.

### 2.2. Pigs

Blood samples for isolation of peripheral blood mononuclear cells (PBMC) and for in vivo experiments were extracted from nine-week-old piglets (*Sus scrofa domestica*), which were obtained by crossbreeding of Hampshire and Yorkshire races. Procedures involving swine were carried out at the Center for Reproduction and Animal Biotechnology (INTA-Maimónides University, Buenos Aires, Argentina) under the supervision of veterinarians.

Blood samples for the PBMC experiments were collected by puncturing the external jugular vein of three pigs by using syringes with sodium citrate as anticoagulant. For the in vivo experiments, five piglets were sedated with xylazine and intravenously immunized by injecting 1 × 10^9^ plaque-forming units (PFU) of AcMNPV in the auricular vein of the ear. Blood samples were collected from the external jugular vein, using syringes with sodium citrate, immediately before the immunization (time zero) as well as at 3, 6, and 9 h post-inoculation (hpi). Plasma was obtained by centrifugation of blood samples (1500× *g* for 10 min), and then conserved at −80 °C for the determination of IFN levels and antiviral activity.

### 2.3. Ethics Statement

All experiments were carried out in accordance with the recommendations in the Guide for the Care and Use of Laboratory Animals of the National Institutes of Health. The animal handling and experimental procedures were approved by our Institutional Experimentation Animal Committee (procedure no. 49/2013). None of the pigs showed clinical signs or signs of suffering during or after inoculations or samplings.

### 2.4. Peripheral Blood Mononuclear Cells

PBMCs were purified from blood samples by Ficoll-Paque density gradient (1.077 g/mL, GE Healthcare) and cultured in DMEM supplemented with FBS 10% (Natocor, Villa Carlos Paz, Córdoba, Argentina), Glutamax (Gibco) and antibiotic/antimycotic solution (Gibco) at 39 ° C for 16 h. For the study of the induction of antiviral soluble factors, 5 × 10^6^ PBMCs seeded in 12-well plates were infected with AcMNPV, Ac-pCpG, or Ac-GVSV at different multiplicities of infection (moi) or with mock of infection and incubated at 39 °C for 18 h. Supernatants were collected, centrifuged at 16,000× *g* for 1 h to eliminate any residuary virion in the pellet, and the supernatants titrated by end-point dilution to confirm the absence of AcMNPV. These supernatants were conserved at −80 °C.

### 2.5. IFN Measurement

IFN-α level was determined in supernatants and serum samples by an in-house ELISA. A 96-well plate (H2B Maxisorp, NUNC) was coated with 100 µL/well of anti-porcine IFN-α mAb (pIFN-α) (clone K9, 1:4000, MBL Company, product number 27100-1, lot number 3095) in carbonate buffer (sodium carbonate/bicarbonate 0.1 M, pH 9.5), and incubated overnight at 4 °C. The plates were washed five times with 100 µL of PBS-Tween (Tween 20 0.05%) (PBS-T) and blocked for 1 h with 200 µL of PBS-T with 5% skin milk and 2% equine normal serum at 37 °C. After five washes, dilutions of the samples and the standard curve (recombinant pIFN-α from PBL Assay Science, catalog number 17105-1, lot number 6481), done in PBS-T with 5% milk and 2% equine normal serum were added in duplicate and the plates were incubated at 37 °C for 2 h. After five additional washes, 100 µL of a dilution (1:5000) of rabbit polyclonal anti-p IFN-α antibody was added and incubated at 37 °C for 1 h. After five washes, a goat anti-rabbit antibody conjugated to horseradish peroxidase (HRP, 1:2500, KPL) was added before incubating the plate at 37 °C for 1 h. Finally, the plates were washed seven times before performing a last incubation with 100 µL of commercial TMB Substrate Reagent Set (BD Biosciences, Thermo Fisher Scientific, Buenos Aires, Argentina) at 37 °C for 30 min in darkness. The reaction was stopped with 50 µL of 2N H_2_SO_4_ and its optical density read at 450 nm in an ELISA reader (Multiskan, Labsystems, Buenos Aires, Argentina).

IFN-γ levels were determined in plasma samples by sandwich ELISA, following the manufacturer’s instructions (Swine IFN gamma ELISA Kit, Invitrogen, catalog number KSC4021, lot number 210634006).

### 2.6. Antiviral Activity Assays

The antiviral activity was measured by treating MDBK or LFBK cells seeded in 96-well plates with two-fold serial dilutions of cell culture supernatants or porcine plasma at 37 °C for 16 h. A standard pIFN-α two-fold dilution curve in duplicate was also included in each plate (recombinant pIFN-α from PBL Assay Science, Piscataway, NJ, USA). For some experiments, samples were pretreated with normal mouse serum (NMS) or with a neutralizing mAb against IFN-α (clone K9). Then, the treated MDBK or LFBK cells were washed and infected with VSV (moi = 0.01) or FMDV (moi = 0.1), respectively, at 37 °C for 24 h. In both assays, cells were fixed with formol 5% and the cytopathic effect was evaluated by staining with crystal violet [57]. The staining was resuspended in a solution of ethanol 50% and acetate 0.1% and the optical density was measured at 595 nm.

The wells displaying an optical density lower than a half of that corresponding to uninfected wells were considered as infected. Based on these results, the half maximal inhibitory concentration (IC_50_) was calculated as the dilution factor that prevented the infection in 50% of the wells. In order to express the results as an absolute value, the units of antiviral activity were calculated by comparison of the IC_50_ of each sample with the IC_50_ of the dilution curve of standard pIFN-α as reference (4.62 × 10^8^ U/mg). The IC_50_ for pIFN-α in the assays was ~7.22 U/mL.

### 2.7. Recombinant AcMNPV Construction

The sequences from the immunostimulatory oligodeoxynucleotides (ODNs) 2216 [58,59], D19 [58,60], H1a [59], and ODN 3 [61] (Table 1) were combined in a single sequence of 75 bp (GGGGGACGATCGTCGGGGGGGGTGCATCGATGCAGGGGGGGGTATTTCGAAATAGGGGGGGCTAGACGTTAGCGT). This sequence repeated six times in tandem (450 bp), called pCpG construct, was chemically synthesized by the company GenScript with an EcoRI restriction site at both extremes and included in the plasmid pUC57.

The “Bac to Bac” system (Invitrogen) was used to construct the recombinant AcMNPV. To this end, the sequence coding for G-VSV (1636 bp) from the commercial plasmid p-CMV-VSV-G (Cell Biolabs, San Diego, CA, USA) or the porcine CpG (pCpG) construct from the plasmid pUC57-pCpG was cloned downstream the polyhedrin promoter of the pFastBacI plasmid (Invitrogen) to generate the transfer plasmids pFB-pCpG and pFB-GVSV. The recombinant pFastBac constructs were transformed individually into *Escherichia coli* DH10Bac cells (Invitrogen) to generate the corresponding recombinant bacmids, according to the manufacturer’s instructions. The inclusion of the pCpG or GVSV constructs was confirmed by PCR. Sf9 cells were transfected with the recombinant bacmid DNA with Cellfectin (Invitrogen) and the recombinant AcMNPV vectors were amplified by repeated passages.

### 2.8. Western Blot

The presence of G-VSV in the outer envelope of Ac-GVSV was evaluated by harvesting AcMNPV-infected Sf9 cells five days post-infection and by purifying the viruses from the supernatants by sucrose cushion ultracentrifugation (1.5 h at 80,000× *g*). Each sample was resolved by 12% SDS polyacrylamide gel electrophoresis and the proteins were then transferred onto nitrocellulose membranes. G-VSV protein was detected using monoclonal anti-VSV glycoprotein antibody (clone P5D4, Sigma-Aldrich, Merck, Buenos Aires, Argentina, 1:1000) and AP-labeled anti-mouse IgG goat antiserum (Sigma, 1:5000). The original Western blot figures can be found in the Supplementary File.

### 2.9. Statistics

IFN measurements and antiviral activities were analyzed by ANOVA using GraphPad Prism software (La Jolla, CA, USA). Comparisons between groups were performed by using Student’s *t*-test or paired-samples *t*-test, whereas comparison of the results with the detection limit in cases in which the levels from controls were undetectable was made by using one-sample *t*-test. Values of *p* < 0.05 were considered significant.

## 3. Results

### 3.1. AcMNPV Elicits IFN-α-Mediated Antiviral Activity in Porcine PBMCs In Vitro

**Figure 1:**
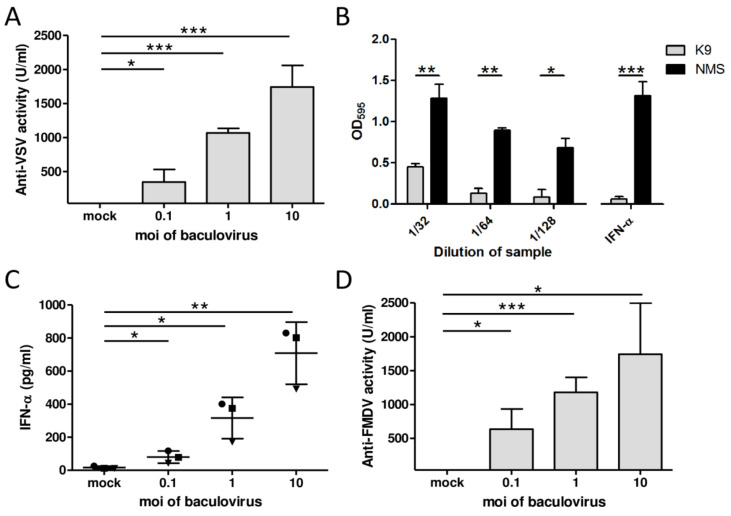
AcMNPV elicits IFN-α-mediated antiviral activity in porcine PBMCs in vitro. PBMCs from three piglets were either mock infected or infected with AcMNPV for 18 h and supernatants were subsequently collected. (**A**) The antiviral activity against VSV serotype Indiana of the supernatants was assayed in MDBK cells. (**B**) Dilutions of supernatants from PBMCs infected with AcMNPV or standard porcine IFN-α (100 U/mL) as control were pretreated with neutralizing anti-IFN-α antibodies or NMS at room temperature for 1 h and assayed for antiviral activity in a bioassay in MDBK cells against VSV infection. The samples were fixed and the assay was revealed with crystal violet; the dye was resuspended and read at 595 nm. (**C**) IFN-α levels were quantified by ELISA. Each symbol represents PBMCs from a distinct pig. (**D**) Antiviral activity against FMDV O1 Campos in the samples was assayed in LFBK cells. Standard porcine IFN-α was used for the quantification of antiviral activity units in A and D (1 U corresponds to 2.16 pg IFN-α; IC_50_ ~ 7.22 U/mL). The results are from an experiment that is representative of at least two independent experiments. One-sample *t*-test was performed to identify differences in antiviral activity between supernatants from AcMNPV- and mock-treated cells. Student’s *t*-test was performed to compare IFN-α between AcMNPV- and mock-treated cells or antiviral activity between samples pretreated with anti-IFN-α K9 mAb and those pre-treated with NMS. * *p* < 0.05; ** *p* < 0.01; *** *p* < 0.001.

Within the innate immune milieu, IFNs are the main factors involved in inducing an antiviral status. To study the impact of AcMNPV on porcine immune cells in terms of the production of this kind of soluble antiviral factors, swine PBMCs purified from blood samples from nine-week-old piglets were selected as an ex vivo model and evaluated IFN production by measuring their antiviral activity. With this aim, two viruses were used: VSV, the most commonly used virus in antiviral assays; and FMDV, due to its veterinary relevance as it was explained in the introduction. These assays were performed in MDBK cells, since they are frequently used in antiviral assays with porcine IFNs and VSV, and LFBK cells, a porcine cell line sensitive to FMDV. The supernatants from AcMNPV-infected PBMCs displayed high antiviral activity against VSV in a dose-dependent manner (average values of 344.9 to 1742.7 U/mL) (Figure 1A). To study whether type I IFNs, the factors responsible for the protection elicited by AcMNPV in mice, mediated the antiviral activity, these supernatants were pretreated with a neutralizing mAb against IFN-α. They displayed significantly lower antiviral activity against VSV than when pretreated with NMS as control (Figure 1B), indicating that IFN-α was responsible for at least part of the protection. Additionally, the strong induction of type I IFNs was evidenced by ELISA analysis of the supernatants (Figure 1C). These results showed that an AcMNPV treatment can induce IFN-α-mediated antiviral activity in porcine PBMCs in a dose-dependent manner.

To test the ability of the supernatants of AcMNPV-infected PBMCs to specifically protect swine cells from an infection with FMDV, porcine LFBK cells were pretreated with serial dilutions of the supernatants and challenged with FMDV O1 Campos. Supernatants transferred from AcMNPV-infected PBMCs showed a very high anti-FMDV effect, with average values that ranged from 634.7 to 1742.4 U/mL depending on the quantity of AcMNPV (Figure 1D).

### 3.2. AcMNPV Induces Type I and II IFNs and Antiviral Activity in Swine

**Figure 2:**
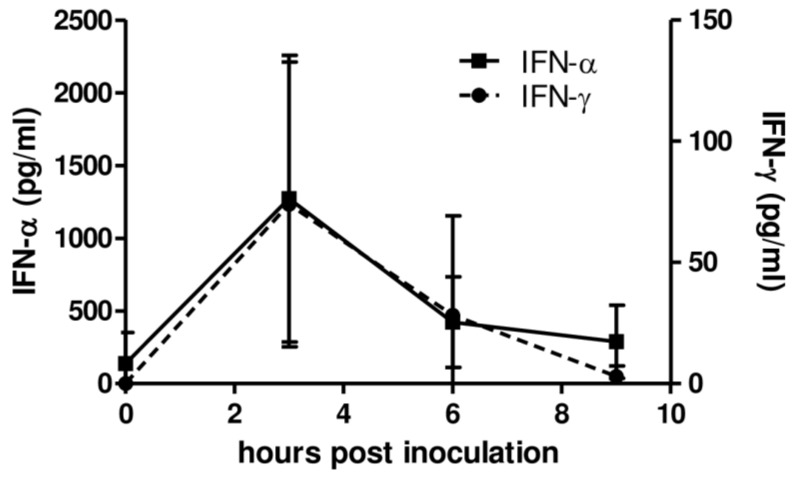
Type I and type II IFNs are detected in sera from AcMNPV-inoculated pigs. Five piglets were intravenously injected with 1 × 10^9^ PFU of AcMNPV and serum samples were collected both before the administration and at 3, 6, and 9 hpi. IFN-α and IFN-γ levels in sera were quantified by ELISA.

These results encouraged us to test the ability of AcMNPV to induce IFNs in pigs in vivo. For this purpose, AcMNPV (1 × 10^9^ PFU) was administered to the animals and collected blood samples at different times to analyze IFN-α and IFN-γ levels in sera by ELISA. The peaks of type I and type II IFN production in plasma were detectable within the first 6 hpi, with a mean of 1364.1 pg/mL and 74 pg/mL for IFN-α and IFN-γ at 3 hpi, respectively (Figure 2).

**Figure 3:**
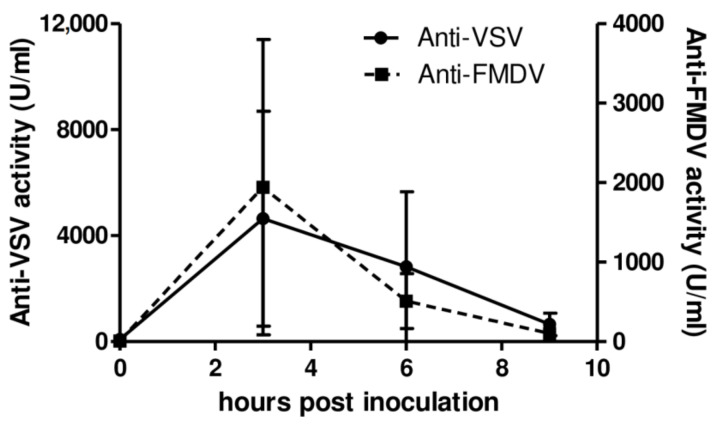
Antiviral activity against VSV and FMDV is detected in sera from AcMNPV-inoculated pigs. Five piglets were intravenously injected with 1 × 10^9^ PFU of AcMNPV and serum samples were collected both before the administration and at 3, 6, and 9 h post-inoculation. The antiviral activity against VSV serotype Indiana in sera was assayed in MDBK cells. The antiviral activity against FMDV O1 Campos in sera was assessed in LFBK cells. In both cases, standard porcine IFN-α was used for the quantification of antiviral activity units (1 U corresponds to 2.16 pg IFN-α; IC_50_ ~ 7.22 U/mL).

Further in vitro assays (Figure 3) revealed that plasma from AcMNPV-inoculated pigs displayed high antiviral activity, inducing protection in porcine non-immune cells against an in vitro challenge with FMDV. Strong antiviral activity was elicited by AcMNPV both against VSV (1547 U/mL in average at 3 hpi) and FMDV (5827.8 U/mL in average at 3 hpi). Altogether, the results showed that AcMNPV inoculation was capable of inducing the production of type I and type II IFNs in pigs in vivo and, therefore, to promote the generation of antiviral activity.

### 3.3. Pseudotyping with G-VSV but Not the Enrichment with pCpG Motifs Improves the Ability of AcMNPV to Induce IFN-α In Vitro

**Figure 4:**
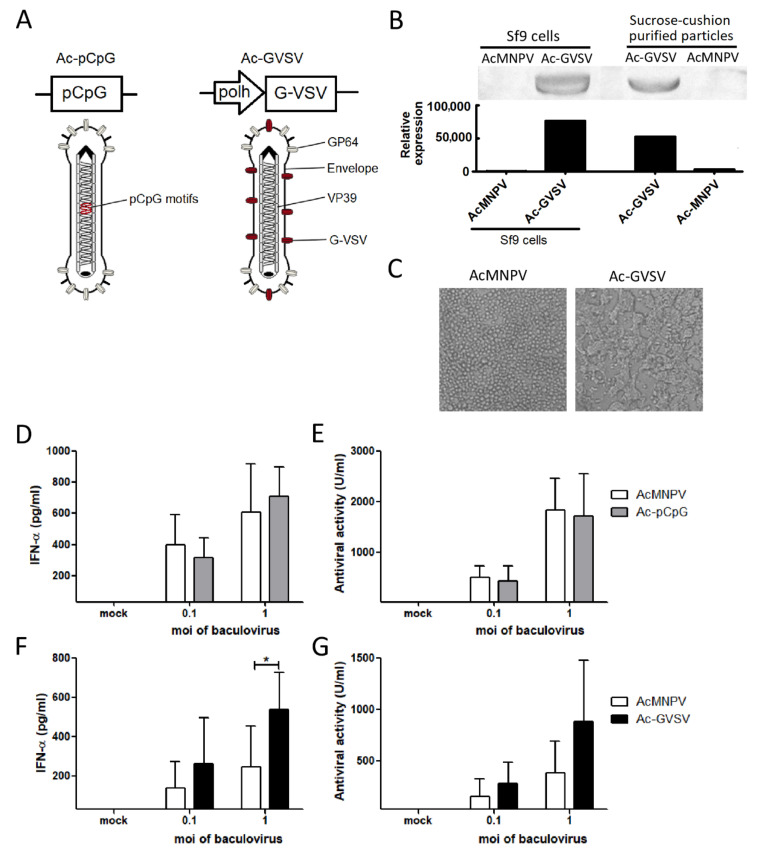
The pseudotyping of AcMNPV with G-VSV, but not the enrichment with immunostimulatory CpG ODNs, improves the induction of IFN-α. (**A**) Schematic representation of the recombinant baculoviruses Ac-GVSV and Ac-pCpG. The main components are pointed out. (**B**) Western blots of sucrose-cushion purified Ac-GVSV particles and AcMNPV- and Ac-GVSV-infected Sf9 cells were revealed with anti-GVSV mAb to evidence the presence of the glycoprotein and the density reading of each band was represented in the graph below. (**C**) Sf9 cells were infected with AcMNPV or Ac-GVSV at 27 °C and pH 6.2 for 7 days and observed by optical microscopy (200×). (**D**–**G**) PBMCs from three piglets were either mock-infected or infected with AcMNPV (3.37 × 10^8^ ± 1.2 × 10^7^ PFU/mL), Ac-pCpG (2.69 × 10^8^ ± 5.1 × 10^7^ PFU/mL), or Ac-GVSV (2.44 × 10^8^ ± 2.55 × 10^7^ PFU/mL) at the indicated moi for 16 h to subsequently collect the supernatants. (**D**,**F**) IFN-α levels in supernatants were quantified by ELISA. (**E**,**G**) The antiviral activity against VSV serotype Indiana of the supernatants was assayed in MDBK cells. Standard porcine IFN-α was used for the quantification of antiviral activity units (1 U corresponds to 2.16 pg IFN-α; IC_50_ ~ 7.22 U/mL). The results are from one experiment that is representative of at least three independent experiments. Paired sample *t*-test was performed in all the experiments to identify differences between viruses. * *p* < 0.05.

Finally, by adopting two different molecular approaches (Figure 4A), the possibility of enhancing the impact of AcMNPV on the induction of type I IFNs in porcine immune cells was assessed. The first approach consisted in enriching the genome of AcMNPV with specific CpG sequences for the porcine TLR9 (Ac-pCpG) (Table 1). The second approach consisted in pseudotyping AcMNPV with the protein G-VSV (Ac-GVSV). A functional G-VSV was present both in Ac-GVSV-infected Sf9 cells and in sucrose cushion ultracentrifuged virions, as evidenced by Western blot (Figure 4B) and by the syncytium formation observed in Sf9 cells infected with Ac-GVSV, but not with AcMNPV, due to the fusogenic activity of the protein (Figure 4C).

Then the previous experiments done in porcine PBMCs were repeated using Ac-pCpG or Ac-GVSV to compare the effect of these recombinant viruses with that of AcMNPV. Low moi of baculoviruses were used in the assays in order to avoid saturation of the response and to be capable of detecting increments in the IFNs induction. No significant differences were detected in the induction of IFN-α (measured by ELISA in the supernatants) or the antiviral activity between AcMNPV and Ac-pCpG (Figure 4D,E). These results indicate that additional CpG motifs do not produce differences in type I IFNs-mediated antiviral activity in the tested conditions. Conversely, pseudotyped Ac-GVSV induced higher levels of IFN-α in comparison to AcMNPV, although the increase in antiviral activity was non-significant (Figure 4F,G). Thus, one of the two genetic engineering approaches evaluated was useful to improve the impact of AcMNPV on the production of type I IFN in porcine cells.

## 4. Discussion

Among the many strategies studied to rapidly contain outbreaks caused by highly contagious viruses in pigs, the administration or expression of porcine IFN-α [17,62,63], β [15], γ [9,14], or λ [16] or the use of some TLR ligands like poly I:C [64] have been some of the most successful ones. On the other hand, the intramuscular inoculation of CpG ODNs, which are TLR-9 ligands, failed in providing protection in pigs against a challenge with FMDV 2 days after inoculation [65].

The use of AcMNPV is a potential safe alternative strategy, owing to its low cytotoxicity and incapacity to replicate or to integrate its genome into vertebrate cells [66]. AcMNPV is able to enter a wide variety of mammalian cell types due to the promiscuity of its surface protein, GP64, which targets very efficiently the genome to different intracellular destinies that are relevant for the induction of innate immunity [29,30,31,32,33,34,35,36,37,38,67]. The impact of its genome on innate immunity sensors [29,36,37,38,39,40,67] induces the production of pro-inflammatory cytokines and IFNs and, consequently, the maturation of dendritic cells [39,42,68,69] and the activation of NK cells in mice [40,69]. Therefore, owing to the strong innate immunity induced in mice, this strategy is very promising. Moreover, as baculoviruses are used as expression vectors, among other applications, their production in insect cells or larvae is standardized, inexpensive, easily scalable, and widely popularized.

In this work, it was firstly demonstrated that the baculovirus AcMNPV induces the production of type I IFNs in porcine PBMCs in vitro. It was also found that IFN-α was at least partially responsible for the high levels of anti-VSV and anti-FMDV activity displayed in the supernatants from AcMNPV-infected porcine PBMCs. These results proved the capacity of AcMNPV to trigger the expression of these antiviral soluble factors in porcine cells.

Despite the lack of studies on the innate immunity induced by baculoviruses in pigs, some authors have successfully tested these viruses as vaccine vectors in this species and found that they elicited humoral and cellular immune responses [51,52,53,54,55]. In this work, the immunostimulatory properties of baculoviruses in pigs were confirmed by showing the in vivo induction of high levels of type I and type II IFNs. Furthermore, the production of IFNs temporally correlated with the anti-VSV and anti-FMDV activity measured in vitro in plasma samples. Although it would be interesting to measure the expression of interferon stimulated genes in target tissues of viral infections some days after inoculation, previous data have shown that IFNs were produced within the first 12 h after inoculation of AcMNPV in mice [39,42], and the levels reached were high enough to establish a protective antiviral status that lasts for several days [48,49,50].

Based on the mechanisms involved in the activation of the murine innate immunity by AcMNPV, in this study, two recombinant AcMNPVs were designed to assess the feasibility to improve the impact of baculoviruses on the generation of antiviral activity. Firstly, the relevance of the TLR9/MyD88-dependent pathway and that the CpG motifs recognized by TLR9 from different species were taken into account [22]. The enrichment of the AcMNPV genome in immunostimulatory CpG sequences with previously proved effects on the porcine immune response [58,59,60,61] failed to produce a measurable improvement in the production of IFN-α and antiviral activity in supernatants from PBMCs. Nonetheless, it cannot be discarded that Ac-pCpG could have a differential effect on specific porcine cell types.

On the other hand, several works have demonstrated that the pseudotyping of AcMNPV with G-VSV improves its transduction efficiency in mammalian cells, with a high effect on the porcine cell line PK15 [27,28]. In this work, it was demonstrated that the pseudotyping with G-VSV also induced an increase in the production of IFN-α in PBMCs, which potentiates the elicited antiviral activity. Moreover, Ac-GVSV could have different effects on other aspects of the immune response such as the maturation of dendritic cells or the activation of NK cells, an issue that deserves further attention.

## 5. Conclusions

This is the first report demonstrating the ability of the baculovirus AcMNPV to elicit the production of IFNs in vitro in PBMCs and in vivo in the swine model. Although more practical routes of injection, doses, and in vivo challenges should be done to establish optimal conditions, we think the results described here using baculoviruses as inductors of antiviral activity in mammals of veterinary interest constitute a proof of concept about the feasibility of the development of a baculovirus-based strategy for the fast control of infectious diseases in swine by the induction of IFNs.

## Figures and Tables

**Table 1:** Sequences of the ODNs selected to enrich the AcMNPV genome in specific CpG motifs for pigs. Four ODNs were selected from studies that demonstrated their immunostimulatory effects in porcine cells. The nucleotide sequences and references are shown in the table.

ODN	Sequence	References
2216	GGG GGA CGA TCG TCGGGGGG	[58,59]
D19	GGT GCA TCG ATG CAGGGGGG	[58,60]
H1a	GGTATTTCGAAATAGGGGGG	[59]
ODN 3	GCT AGA CGT TAG CGT	[61]

## Data Availability

The data presented in this study are available on request from the corresponding author.

## Supplementary Materials

The following are available online at https://www.mdpi.com/article/10.3390/vetsci8110278/s1, Original Western blot figures.
