# Supplementary material for: Baculovirus Vectors Induce the Production of Interferons in Swine: Their Potential in the Development of Antiviral Strategies
Source: Vet Sci. 2021 Nov 17;8(11):278. doi: 10.3390/vetsci8110278 (PMC8617851; doi:10.3390/vetsci8110278)
Supplement: Supplementary file 1 [file vetsci-08-00278-s001.zip › vetsci-1366577-supplementary.pdf]

cancers

Supplementary Materials

Baculovirus Vectors Induce the Production of Interferons in Swine: Their Potential in the Development of Antiviral Strategies

Guido Nicolás Molina, Sabrina Amalfi, Ignacio Otero, Oscar Taboga, and María Paula Molinari

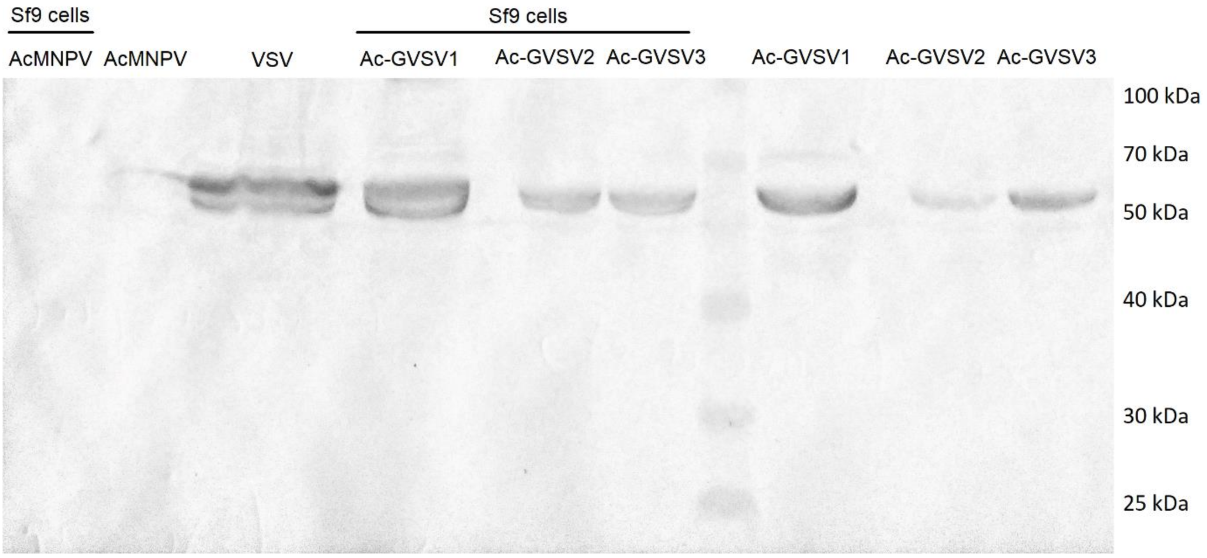

Original Western blot figures.
